# Chest Computed Tomography Findings in COVID-19 and Influenza: A Narrative Review

**DOI:** 10.1155/2020/6928368

**Published:** 2020-06-05

**Authors:** Stephen O. Onigbinde, Ademola S. Ojo, Linwald Fleary, Robert Hage

**Affiliations:** ^1^Department of Anatomical Sciences, St. George's University School of Medicine, St. George's, West Indies, Grenada; ^2^Radiology, General Hospital, St. George's, West Indies, Grenada

## Abstract

**Objective:**

The COVID-19 pandemic and annual influenza epidemic are responsible for thousands of deaths globally. With a similarity in clinical as well as laboratory findings, there is a need to differentiate these two conditions on chest CT scan. This paper attempts to use existing literature to draw out differences in chest CT findings in COVID-19 and influenza.

**Methods:**

A search was conducted using PubMed. 17 original studies on chest CT findings in COVID-19 and influenza were identified for full-text review and data analysis. *Findings*. COVID-19 and influenza share similar chest CT findings. The differences found show that COVID-19 ground-glass opacities are usually peripherally located with the lower lobes being commonly involved, while influenza has a central, peripheral, or random distribution usually affecting the five lobes. Vascular engorgement, pleural thickening, and subpleural lines were reported in COVID-19 patients. In contrast, pneumomediastinum and pneumothorax were reported only in studies on influenza. *Conclusion and Relevance*. COVID-19 and influenza have overlapping chest CT features with few differences which can assist in telling apart the two pathologies. Additional studies are needed to further define the differences and degree between COVID-19 and influenza.

## 1. Introduction

On December 31, 2019, the first report emerged of an outbreak of an acute respiratory infection of unknown origin in Wuhan, Hubei province of China. This highly contagious viral infection caused by the novel coronavirus (2019-nCoV or SARS-CoV-2) rapidly spread across the country [1, 2]. By January 29, 2020, the number of confirmed cases had increased to 5997 in China with 132 deaths and 68 cases reported in 15 other countries [3]. The World Health Organization declared 2019-nCoV a global pandemic by March 12. The virus rapidly swept through countries with 1,773,084 confirmed cases and 111,652 deaths across 210 countries and territories by April 13, 2020 [4].

Prior to the current 2019-nCoV pandemic, there have been multiple episodes of global outbreak of acute respiratory disease caused by various strains of influenza virus. The most recent episode was the 2009 influenza A (H1N1) pandemic which spread over 214 countries between March 2009 and August 2010 resulting in 18,449 laboratory-confirmed fatalities globally [5]. While the influenza pandemic might be said to be over, the H1N1 as well as other strains of influenza virus have remained with us as seasonal viruses, resulting in the annual seasonal influenza epidemics [6]. Influenza occurs globally affecting 5-10% of adults and 20-30% of children annually with most cases occurring during the winter months in the northern (November to April) and southern hemispheres (April to September) with no seasonal pattern in the tropical regions [7, 8]. Influenza-related respiratory diseases are responsible annually for an estimated 650,000 deaths globally [9]. With the current global focus on the 2019-nCoV outbreak, the threat from the existence of the equally contagious influenza virus with seasonal manifestations cannot be downplayed.

SARS-CoV-2 and influenza primarily affect the respiratory system. SARS-CoV-2 gains access to the host cells using its surface spike (S) protein to bind human angiotensin-converting enzyme 2 receptor on host cells. The viral entry triggers a host immune response with a massive release of cytokines thought to be responsible for organ dysfunctions [10]. Influenza enters the epithelial cells and alveolar macrophages using two surface proteins, hemagglutinin which binds to sialic acid-containing molecules and neuraminidase which cleaves sialic acid to facilitate viral entry into host cells [11]. An inflammatory response follows with the recruitment of neutrophils, macrophages, and other immune cells. While this response is important for viral clearance, it also plays a role in various disease manifestations [12].

Most cases of 2019-nCoV infection are asymptomatic or present with mild symptoms. In a series of 72,314 2019-nCoV cases published by the Chinese center for disease control, only 19% of cases had severe or critical disease with a case fatality rate of 2.3% [13]. The common symptoms manifested by affected individuals are fever, fatigue, cough, and sputum production. Sore throat, muscle pain, and gastrointestinal symptoms including nausea, vomiting, and diarrhea were less commonly reported [14–17]. This is similar to the clinical picture in influenza with fever, cough, and sore throat being often mentioned [18]. The reported findings from laboratory studies in individuals with 2019-nCoV include leukopenia, lymphopenia, elevated C-reactive protein, elevated lactate dehydrogenase, elevated aspartate transaminase, and prolonged prothrombin time [14, 19]. This is similar to the laboratory reports for cases of influenza [20]. Although these findings are not diagnostic of 2019-nCoV or influenza infection, they correlate with the risk of increased severity such as the development of acute respiratory distress syndrome and death [21, 22].

The gold standard for the diagnosis of these viral infections is the confirmation of viral RNA by real-time reverse transcriptase polymerase chain reaction (RT-PCR). However, based on a previous report on 2019-nCoV, the positive rate of RT-PCR at initial presentation is 30-60%. This may be due to a low viral load, hence the need for repeated testing [23, 24]. The available kits for testing influenza viruses reportedly have a sensitivity of 66-100% [25]. Although not a substitute for RT-PCR in the diagnosis of COVID-19 and influenza, chest computed tomography (CT) has been found to have an increasing use in the management of viral pneumonia [24].

Other imaging modality such as plain chest radiograph is useful in the evaluation of many chest disorders including viral chest infections. It however fails in the detection of early interstitial diseases. Ultrasonography has been employed in the detection of interstitial disorders, consolidation, and effusions [26]. CT scans, however, have a higher resolution and a greater ability to provide detailed anatomy, making it a superior tool in the evaluation of patients with 2019-nCoV and influenza. There is a need to clearly differentiate between these two conditions on imaging due to the similarities in clinical symptomatology as well as laboratory findings, in order to give focused care targeted at each condition. This study therefore focuses on a review of literature to assess the differentiating chest CT findings in 2019-nCoV infection and influenza.

## 2. Methodology

This study is based on a review of published literature on the chest CT findings in COVID-19 and influenza. Literature search was done using PubMed to find MEDLINE indexed articles relevant to this study. The search was conducted using the keywords ‘COVID-19', ‘SARS-CoV-2', ‘chest CT scan', ‘influenza', ‘pandemic', or any combination of these terms. The search terms yielded 435 studies. Using abstracts, two investigators independently selected articles for full-text review. Discrepancies were jointly reviewed and decided upon.

The inclusion criteria used were (1) original studies in the form of case series, cohort, and retrospective studies; (2) a sample size of at least 10; (3) studies conducted in adult patients (≥18 years); (4) written in English; and (5) carried out since the onset of the 2009 global influenza pandemic till date. Exclusion criteria included (1) editorials and studies with unavailable raw data, (2) repeated published articles, and (3) case reports. The full texts of 17 selected studies were reviewed. Nine were COVID-19 studies and eight were articles on influenza. This resulted in reviewing 347 CT findings for COVID-19 and 743 for influenza.

Demographic information such as sample size, mean age, and range was tabulated. Frequency tables of the chest CT findings were established using the following categories: parenchymal features, distribution of parenchyma features, bronchovascular changes, pleural involvement, and extrapleural findings. Features not found were represented as “0,” while unreported features were indicated by “-.”

## 3. Results

A total of 17 studies, made up of 9 studies on COVID-19 and 8 on influenza, formed the basis of the comparison. The number of cases ranges from 11 to 149 and 10 to 135 in COVID-19 and influenza, respectively. The mean age in both set of studies is similar with most lying between 40 and 50 years (Table 1). The most common parenchyma lesions reported in both studies are ground-glass opacities (GGO) found in 12.1-100% in all COVID-19 studies and 33.3%-95% in all influenza studies, followed by consolidation, reported in 7.2-54.5% in 6 COVID-19 studies and 14.3-93% in 6 influenza studies (Table 2). A mixed pattern of GGO with consolidation was found in 26.8-64.4% in 5 COVID-19 studies and 30-58% in 3 influenza studies. Nodules were reported in 2-27.3% in 4 COVID-19 studies and 4.8-66% in 4 influenza studies. A crazy-paving pattern reported was 12-70.6% in 5 COVID-19 studies and 15-40% in 3 influenza studies. A reticular pattern was found as 48.5-81.8% in 4 COVID-19 studies and in 11.9-20% in 2 influenza studies (Table 2). Other parenchyma findings are interlobular septal thickening, reported in 35-70.6% in 3 COVID-19 studies and 5-21% in 3 influenza studies, and linear opacity, found in 18.2-61% in 2 COVID-19 studies.

The predominant distribution of lesions is multifocal, reported in 53-83.9% in 4 COVID-19 studies and >50% in 2 influenza studies, multilobar, with 5 COVID-19 studies reporting more than 1 lobar involvement in 59-75% of cases and 1 study on influenza reporting multilobar predominance in 75% of cases. Most are bilaterally distributed, found in 59-82.2% in 3 COVID-19 studies and 71-100% in 5 influenza studies. All COVID-19 studies found a peripheral predominance of distribution in 35.9-100% of cases, while 2 influenza studies reported a peripheral distribution in 55-65% of cases and 2 studies reported central distribution in 44.3-60% of cases. The lower lobe was the predominantly affected lobe in 5 COVID-19 and 3 influenza studies (Table 3).

Bronchovascular changes reported in the studies include air bronchogram reported as 8-72.7% of 7 COVID-19 studies and none in influenza studies. Peribronchovascular predominance of lesions was reported as 10-75% in 5 COVID-19 studies only. Bronchial dilatation was found in 11-52.5% in 3 COVID-19 and 45% in one study on influenza. Vascular engorgement was reported as 71.3-82.4% in 4 COVID-19 studies only. Tree-in-bud appearance was found only in one study on COVID-19, 9.1%; none was reported in the influenza studies (Table 4).

## 4. Discussion

Clinical differentiation of COVID-19 from influenza is challenging because both diseases have an asymptomatic period of just over a week. During this asymptomatic period, the infection can be transmitted [22]. In a recent study, radiologists could differentiate COVID-19 from other viral pneumonia about 74% of the time on chest CT images [24]. In this review, chest CT features of COVID-19 were compared to those of influenza [24]. Differences were observed in each of the following categories: parenchymal features and their distribution, bronchovascular changes, and pleural and extrapleural findings.

GGO are hazy areas of increased lung density that do not obscure bronchial and vascular markings [42]. It can be due to partial filling of airspaces or interstitial thickening [34]. Previous studies have identified GGO as the earliest and predominant CT abnormality in COVID-19 patients [16]. A recent study found that GGO was predominantly unilateral, multifocal, and peripheral in preclinical patients but became bilateral and more diffuse in the first week after the onset of symptoms [31]. In another study, GGO increased in opacity within 1-3 weeks to become consolidated [16]. The common findings on CT for typical influenza pneumonia consist of diffuse or multifocal ground-glass opacities and small centrilobular nodules [15]. In this study, the occurrence of ground-glass opacification and consolidation in COVID-19 were not markedly different from that of influenza studies. However, they were predominantly located bilaterally within the lower lobes for COVID-19 patients, while in the influenza studies, they were more widespread, involving all the lobes. This is similar to a recent study in which the GGO of COVID-19 patients were found predominantly peripheral (i.e., outer one-third). The occurrence of GGO and consolidation in this study contrasted with a recent comparative study which reported COVID-19 patients had significantly higher probability of ground-glass opacity appearance relative to other viral pneumonias (91 vs. 68%, *p* < 0.001) [24]. This is likely due to differences in time-to-imaging.

Consolidation is opacification that occurs by pathological tissues, cells, or fluid replacing the alveolar cells, obscuring the underlying vessels [42, 43]. It can be segmental or subsegmental [44]. Consolidation has been reported as the second predominant feature in COVID-19 patients within the first few days of disease onset, the presence of which could indicate an increase in disease severity [42].

The crazy-paving pattern manifests with thickened interlobular septal and intralobular lines superimposed on ground-glass opacity [6, 42, 45]. Crazy pattern and reticular changes were more reported in COVID-19 studies. A previous study reported a higher likelihood of reticular changes in COVID-19 patients [24]. It was noted that fine reticular opacities in COVID-19 patients appear with a ratio of 56 vs. 22% (*p* value < 0.001) which is a result of the thickening of the pulmonary interstitial structures such as interlobular lines and interlobular septa [17, 30, 33]. The presence of the crazy-paving pattern could indicate an advanced disease stage, as it was the main finding in the third week after the onset of symptoms in a recent study [31].

A nodule is a round or irregular opacity of less than 3 cm in diameter with sharp or ill-defined margins. They are classified as centrilobular or “tree-in-bud” nodules and discrete focal nodules [6]. Nodules, particularly centrilobular nodules, are less commonly seen with pneumonia-like infections such as COVID-19 and influenza [46]. This is similar to findings in this review. Bacterial superinfection may be suggested when patients have pleural effusion, extensive tiny lung nodules, and lymphadenopathy [42].

Pleural changes can either be reported as pleural effusion or pleural thickening. Pleura effusion is the filling of the pleural space with fluid [47]. It could be transudative-normal pleural or exudative-fluid from infection [47]. In this review, pleural effusion was rarely found with COVID-19 (Table 5). This is similar to findings in other studies [30, 48]. It occurs less frequently than pleural thickening. The presence of pleural effusion may suggest a poor prognosis in COVID-19 patients [17]. Pleural thickening is a process where the pleura is thickened, usually with scar tissue [47], and it could be caused by acute inflammation of the pleura. The subpleural line is a thin curvilinear opacity of about 1-3 mm in thickness found close to the pleural surface [12]. It is located in the subpleural region and distributed parallel to the pleural surface. Pleural thickening was observed only in COVID-19 cases. Similarly, subpleural thickening was also reported only in COVID-19 studies (Table 5). The peripheral location of ground-glass opacities in COVID-19 patients may be a contributory factor for the relative increase of pleural and subpleural thickening identified on images.

Pneumothorax is air within the pleural space, while pneumomediastinum is air within the mediastinum. They are rarely seen in COVID-19 and influenza. The most common cause of pneumomediastinum is rupture of the alveoli, due to coughing, vomiting, straining, or blunt chest trauma [49]. Free air may dissect through the visceral pleura causing pneumothorax or track centrally to the hilum and mediastinum causing pneumomediastinum [38]. Pneumothorax and pneumomediastinum were only reported in influenza studies reviewed. In a recent study, all the patients with pneumomediastinum needed extracorporeal membrane oxygenation and advanced mechanical ventilation [41]. The threshold for mediastinal lymphadenopathy is a short axis diameter of 1 cm [50]. The occurrence of lymphadenopathy in the studies reviewed was low and relatively lower in COVID-19 studies. This is similar to findings by a recent study which observed lymphadenopathy in 2.7% of COVID-19 patients and 10.0% of non-COVID-19 patients (*p* < 0.001) [24]. However, the presence of lymphadenopathy is considered a significant risk factor for severe COVID-19 pneumonia; this may be due to bacterial superinfection [31].

## 5. Conclusion

This review compared chest CT findings of COVID-19 and H1N1 influenza cases using existing publications. The differences found show that COVID-19 ground-glass opacities are usually peripherally located compared with influenza which also had central and random locations. Vascular engorgement, pleural thickening, and subpleural lines were more frequently reported in COVID-19 patients. Lymphadenopathy was rare in both COVID-19 and influenza patients. In contrast, pneumomediastinum and pneumothorax were reported only in studies on influenza.

### 5.1. Limitations

There are limitations to this review. Firstly, COVID-19 is relatively novel and thus the studies are limited in number. The CT findings reported about influenza were before the onset of COVID 19. A direct comparison of pictures now may better define the differences. Secondly, the review could not accurately account for the differences in time to imaging relative to the onset of symptoms. Thirdly, this review cannot account for findings due to the use of various machine models and imaging protocols. Finally, the use of abstracts in selecting full texts for a detailed review could have led to the omission of some articles. A meta-analysis is recommended to further define the differences and the degree between COVID-19 and influenza.

## Figures and Tables

**Table 1:** Demographic characteristics and number of CT scans done.

Author	Study group	Sample size	Mean age/range (years)	Number of CT scans
Xu et al. [27]	C	90	(37-78)	Multiple
Yang et al. [28]	C	149	45.0	Multiple
Wang et al. [29]	C	90	45.0	Multiple
Lu et al. [16]	C	11	50.0	Single
Zhou et al. [30]	C	62	52.8 (30-77)	Multiple
Shi et al. [31]	C	81	49.5	Multiple
Zhao et al. [32]	C	101	44.4 (17-75)	Single
Han et al. [17]	C	108	45.0 (21-90)	Single
Li and Xia [33]	C	51	58.0 (26-83)	Single
Amorim et al. [34]	I	71	41.3 (16-92)	Single
Elicker et al. [35]	I	20	48.2	Multiple
Henzler et al. [36]	I	10	44.1	Single
Jartti et al. [37]	I	135	46.8 (25-74)	Single
Marchiori et al. [38]	I	20	42.7 (24-72)	Single
Nicolini et al. [39]	I	28	53.8	Single
Shim et al. [40]	I	21	37.0 (6-82)	Single
Valente et al. [41]	I	42	40.9 (21-76)	Multiple

C: COVID-19; I: influenza.

**Table 2:** Parenchymal changes.

Author	Study group	GGO, *N* (%)	Consolidation, *N* (%)	GGO+consolidation, *N* (%)	Nodules, *N* (%)	Crazy-paving appearance, *N* (%)	Linear opacity, *N* (%)	Interlobular septal thickening, *N* (%)	Reticular pattern, *N* (%)
Xu et al. [27]	C	65 (72.0)	12 (13.0)	—	—	11 (12.0)	55 (61.0)	33 (37.0)	48 (53.0)
Yang et al. [28]	C	18 (12.1)	11 (7.2)	40 (26.8)	3 (2.0)	—	—	—	79 (53.0)
Wang et al. [29]	C	41 (45)-55 (62)	—	—	—	22 (24.0)	—	—	—
Lu et al. [16]	C	11 (100.0)	6 (54.5)	7 (63.6)	3 (27.3)	—	2 (18.2)	—	73 (81.8)
Zhou et al. [30]	C	25 (40.3)	21 (33.9)	—	—	—	—	—	—
Shi et al. [31]	C	53 (65.0)	24 (30)	—	4 (6.0)	8 (10.0)	—	28 (35.0)	—
Zhao et al. [32]	C	87 (86.1)	44 (43.6)	65 (64.4)	—	—	—	—	49 (48.5)
Han et al. [17]	C	65 (60.0)	—	44 (41.0)	—	43 (40.0)	—	—	—
Li and Xia [33]	C	28 (54.9)	—	28 (54.9)	11 (21.5)	36 (70.6)	—	36 (70.6)	—
Amorim et al. [34]	I	60 (85.0)	45 (64.0)	41 (58.0)	18 (25.0)	11 (15.0)	—	15 (21.0)	—
Elicker et al. [35]	I	13 (65.0)	17 (85.0)	—	8 (40.0)	—	—	—	—
Henzler et al. [36]	I	9 (90)	8 (80)	—	—	4 (40.0)	—	—	2 (20.0)
Jartti et al. [37]	I	100 (74.0)	126 (93.0)	—	24 (18.0)	—	—	—	—
Marchiori et al. [38]	I	12 (60.0)	2 (10.0)	6 (30.0)	0	—	—	—	—
Nicolini et al. [39]	I	24 (84.5)	—	—	—	7 (25.0)	—	—	—
Shim et al. [40]	I	20 (95.0)	6 (29.0)	—	3 (14.0)	—	—	1 (5.0)	—
Valente et al. [41]	I	14 (33.3)	6 (14.3)	22 (52.4)	2 (4.8)	—	—	3 (7.10)	5 (11.9)

*N*: number of cases: C: COVID-19; I: influenza; GGO: ground-glass opacity.

**Table 3:** Predominant location of parenchymal findings.

Author	Study group	Multifocal distribution, *N* (%)	No. of lobes, *N* (%)	Bilateral distribution, *N* (%)	Peripheral or central, *N* (%)	Upper/middle/lower
Xu et al. [27]	C	MF 62 (69.0)	>1 lobe 53 (59.0)	B 53 (59.0)	P 46 (51.0)	L
Yang et al. [28]	C	—	—	—	P 53 (35.9)	L
Wang et al. [29]	C	—	>1 lobe	B	P	—
Lu et al. [16]	C	MF 9 (83.9)	>1 lobe	—	P 11 (100.0)	L
Zhou et al. [30]	C	—	—	—	P 48 (77.4)	—
Shi et al. [31]	C	MF 43 (53.0)	—	B 64 (79.0)	P 44 (54.0)	L
Zhao et al. [32]	C	MF 55 (54.5)	>1 lobe 66 (65.0)	B 83 (82.2)	P 88 (87.1)	L
Han et al. [17]	C	—	—	—	P 97 (90.0)	—
Li and Xia [33]	C	—	5 lobes 38 (75.0)	—	P 49 (96.1)	—
Amorim et al. [34]	I	—	—	B 63 (89.0)	—	R
Elicker et al. [35]	I	—	—	B 20 (100.0)	—	—
Henzler et al. [36]	I	—	—	—	R	R
Jartti et al. [37]	I	—	5 lobes 101 (75.0)	—	P 74 (55)	L
Marchiori et al. [38]	I	—	—	B 20 (100.0)	P 13 (65.0)	—
Nicolini et al. [39]	I	MF > 14 (>50.0)	—	B	—	L
Shim et al. [40]	I	MF > 11 (>50.0)	—	—	Ce 12 (60.0)	L
Valente et al. [41]	I	—	—	—	Ce 18 (44.3)	—

*N*: number of cases; C: COVID-19; I: influenza; MF: multifocal; B: bilateral; R: random, Ce: central; P: periphery; L: lower.

**Table 4:** Bronchovascular findings.

Author	Study group	Peribronchovascular, *N* (%)	Air bronchogram, *N* (%)	Bronchial dilatation, *N* (%)	Vascular engorgement, *N* (%)	Tree-in-bud, *N* (%)
Xu et al. [27]	C	—	7 (8.0)	—	—	—
Yang et al. [28]	C	—	81 (54.4)	—	—	—
Wang et al. [29]	C	—	—	—	—	—
Lu et al. [16]	C	—	8 (72.7)	3 (27.3)	—	1 (9.1)
Zhou et al. [30]	C	—	45 (72.6)	—	35 (56.5)	—
Shi et al. [31]	C	—	38 (47.0)	9 (11.0)	—	0
Zhao et al. [32]	C	—	—	53 (52.5)	72 (71.3)	—
Han et al. [17]	C	—	52 (48.0)	—	86 (80.0)	—
Li and Xia [33]	C	—	35 (68.6)	—	42 (82.4)	—
Amorim et al. [34]	I	8 (11.0)	—	—	—	—
Elicker et al. [35]	I	3 (12.5)	—	9 (45.0)	—	—
Henzler et al. [36]	I	—	—	—	—	—
Jartti et al. [37]	I	96 (71.0)	—	—	—	—
Marchiori et al. [38]	I	—	—	—	—	—
Nicolini et al. [39]	I	2.8 (10.0)	—	—		
Shim et al. [40]	I	16 (75.0)	—	—	—	—
Valente et al. [41]	I	—	—	—	—	—

*N*: number of cases; C: COVID-19; I: influenza.

**Table 5:** Pleural and extrapleural findings.

Author	Study group	Pleural effusion, *N* (%)	Pleural thickening, *N* (%)	Lymphadenopathy, *N* (%)	Pericardial effusion, *N* (%)	Pneumomediastinum, *N* (%)	Pneumothorax, *N* (%)	Subpleural line, *N* (%)
Xu et al. [27]	C	4 (4.0)	50 (56.0)	1 (1.0)	1 (1.0)	—	—	—
Yang et al. [28]	C	10 (6.7)	—	7 (4.7)	—	—	—	—
Wang et al. [29]	C	6 (7.0)	—	0	—	0	0	—
Lu et al. [16]	C	—	—	0	0	—	—	—
Zhou et al. [30]	C	6 (9.7)	30 (48.4)	—	—	—	—	21 (33.9)
Shi et al. [31]	C	4 (5.0)	26 (32.0)	4 (6.0)	—	—	—	—
Zhao et al. [32]	C	14 (13.9)	—	1 (1)	—	—	—	28 (27.7)
Han et al. [17]	C	0	0	0	—	—	—	—
Li and Xia [33]	C	1 (1.0)	—	0	—	—	—	—
Amorim et al. [34]	I	19 (27.0)	—	0	—	—	—	—
Elicker et al. [35]	I	7 (35.0)	—	1 (5.0)	3 (15.0)	—	—	—
Henzler et al. [36]	I	9 (90.0)	—	1 (10.0)	—	—	—	—
Jartti et al. [37]	I	8 (6.0)	—	—	17 (12.5)	—	—	—
Marchiori et al. [38]	I	5 (25.0)	—	0	—	—	—	—
Nicolini et al. [39]	I	6 (21.0)	—	0	—	—	—	—
Shim et al. [40]	I	6 (29.0)	—	—	—	5 (24.0)	—	—
Valente et al. [41]	I	7 (16.7)	—	6 (14.3)	—	1 (2.4)	2 (5.0)	—

*N*: number of cases; C: COVID-19; I: influenza.
